# Trends in the prevalence of microscopically-confirmed schistosomiasis in the South African public health sector, 2011–2018

**DOI:** 10.1371/journal.pntd.0009669

**Published:** 2021-09-16

**Authors:** Liesl De Boni, Veerle Msimang, Alex De Voux, John Frean

**Affiliations:** 1 South African Field Epidemiology Training Programme, Johannesburg, South Africa; 2 University of the Witwatersrand, Johannesburg, South Africa; 3 Centre for Emerging Zoonotic and Parasitic Diseases, National Institute for Communicable Diseases, National Health Laboratory Service, Johannesburg, South Africa; NIPD: National Institute of Parasitic Diseases, CHINA

## Abstract

**Background:**

Schistosomiasis, also known as bilharzia, is a chronic parasitic blood fluke infection acquired through contact with contaminated surface water. The illness may be mild or can cause significant morbidity with potentially serious complications. Children and those living in rural areas with limited access to piped water and services for healthcare are the most commonly infected. To address the prevalence of the disease in parts of South Africa (SA) effective national control measures are planned, but have not yet been implemented. This study aimed to estimate the prevalence and trends of public sector laboratory-confirmed schistosomiasis cases in SA over an eight-year (2011–2018) period, to inform future control measures.

**Methodology & principal findings:**

This is a descriptive analysis of secondary data from the National Health Laboratory Service (NHLS). The study included all records of patients for whom microscopic examination detected *Schistosoma* species eggs in urine or stool specimens from January 2011 to December 2018. Crude estimates of the prevalence were calculated using national census mid-year provincial population estimates as denominators, and simple linear regression was used to analyse prevalence trends. A test rate ratio was developed to describe variations in testing volumes among different groups and to adjust prevalence estimates for testing variations.

A total number of 135 627 schistosomiasis cases was analysed with the highest prevalence observed among males and individuals aged 5–19 years. We describe ongoing endemicity in the Eastern Cape Province, and indicate important differences in the testing between population groups.

**Conclusion:**

While there was no overall change in the prevalence of schistosomiasis during the analysis period, an average of 36 people per 100 000 was infected annually. As such, this represents an opportunity to control the disease and improve quality of life of affected people. Laboratory-based surveillance is a useful method for reporting occurrence and evaluating future intervention programs where resources to implement active surveillance are limited.

## Author summary

This is the first paper to describe the prevalence of human schistosomiasis in South Africa using nationally representative data. The prevalence remained consistent during the eight-year period, independent of increasing annual testing volumes. Groups with the highest burden included males and individuals aged 5–19 years. The schistosomiasis-endemic provinces of Limpopo, Mpumalanga and KwaZulu-Natal emerged as the highest-burdened areas, and ongoing endemicity in Eastern Cape Province was demonstrated. Western Cape Province likely reported cases imported from endemic provinces rather than being acquired by local transmission. In summary, schistosomiasis remains an important public health problem in South Africa that needs long-term sustainable, effective standardised interventions to reduce the burden. Passive laboratory-based surveillance is a practical tool for reporting prevalence, and could be used to monitor and evaluate future intervention programs.

## Introduction

Schistosomiasis, also known as bilharzia, is a chronic parasitic disease predominantly found in tropical and sub-tropical African regions [1–3]. The disease is an important public health problem in sub-Saharan Africa, South America, Caribbean, Middle East and Asia because of the associated morbidity and mortality. It is estimated that 280 000 deaths per annum occur due to liver fibrosis, internal haemorrhage, or urinary system complications, caused by schistosomiasis [3–5]. Among the parasitic diseases, it ranks second as a global cause of death [3–5], after malaria, which causes approximately 409 000 deaths per annum [6,7]. Though mortality from schistosomiasis might seem relatively low in the global context, it has been shown to significantly reduce quality of life. Up to 70 million disability-adjusted life years (DALYs) are estimated to be lost annually due to schistosomiasis [8]. This is similar to the DALYs lost through HIV/AIDS and is higher than those of malaria and tuberculosis [9]. The worst affected are poor rural and marginalised communities [5,10] and school-age children [2,11,12]. The propensity of schistosomiasis to affect vulnerable communities should make it a public health priority.

The geographic distribution of human schistosomiasis is limited to areas that provide suitable habitats for certain snail species, which are the intermediate hosts of the parasitic worm. This includes the warmer northern and eastern sections of South Africa, parts of the North West and Gauteng provinces, most of Limpopo, Mpumalanga and KwaZulu-Natal provinces, and parts of the Eastern Cape Province [13–16]. The predominant species found in South Africa is *Schistosoma haematobium*, which causes urogenital schistosomiasis, but infections with *Schistosoma mansoni*, which causes intestinal schistosomiasis, are also observed in certain areas [1–3,5,10–12]. Infection is acquired when people come into contact with fresh water, e.g. lakes, rivers or dams that are infested with suitable infected snail hosts. Infective cercariae emerge from the intermediate snail host to infect people percutaneously and then migrate to certain predilection sites where they mature into adult worms [1,2]. The adult worms reside inside the veins of the urogenital (*S*. *haematobium*) and digestive (*S*. *mansoni*) tracts, where they lay eggs. To be excreted via urine or faeces, the eggs migrate through the tissues into the bladder or colon. Most of the eggs become trapped in various tissues, and it is the resulting inflammatory reaction of the urogenital tract, intestine, or other organs that can cause serious illness [1,2,4]. The urogenital form is characterised by blood in the urine, difficulty urinating, bladder or kidney disease and female genital tract involvement. Signs of the intestinal form include diarrhoea, blood in the stool, liver or spleen enlargement, and portal hypertension (and its complications) [3–5]. Schistosomiasis has been linked with other problems in children including impaired cognitive development [8,17] and typhoid fever relapses [1]. The urogenital form is implicated in poor reproductive health outcomes in women and bladder neoplasia [2,18], and it may increase the risk of HIV acquisition and transmission among women [3,18–20].

Disease surveillance for schistosomiasis in South Africa is passive and relies on disease notifications. In 2017, it was classified as a category two notifiable medical condition (NMC). Category 2 NMCs are required to be notified to the Department of Health, in writing or electronically, within seven days of diagnosis by a health care provider as well as by all diagnostic laboratories [21]. Schistosomiasis is probably underreported because the diagnosis depends on laboratory confirmation, which in turn relies on infected individuals seeking healthcare. People might undergo screening tests as part of independent school-based control programs in endemic areas, but these are presumably few since no formal government-run program is currently in place [3,22]. The addition of schistosomiasis to the list of NMCs is recent [21], and many health care providers are still unaware of the reporting requirements. Thus, reporting remains largely laboratory-based. In general, schistosomiasis prevalence is underestimated as cases may go unreported, due to barriers such as low disease awareness, minimal symptoms, or cases not presenting because of poor healthcare access in rural areas where the disease prevails [10,23]. Another contributing factor to South Africa’s underestimation of the prevalence is that laboratory data were previously managed and stored separately at provincial level. A new centralised national electronic laboratory information system (LIS) was implemented in 2010, starting with the provinces without any LIS. A gradual change over from the former to the new LIS was completed in the remaining provinces by 2016. Thus, national-level laboratory data for the public health sector was first accessible starting in 2010.

According to the national guidelines for primary health care, accessed by 73–87% of South Africans [24], all patients with haematuria or suspected of having schistosomiasis should be tested by having a urine or stool specimen examined microscopically [25]. The prescribed medication for the infection, praziquantel [25], is safe and effective [3,26,27]. Empirical treatment in endemic areas is recommended if microscopy testing is not possible and glomerulonephritis has been ruled out as a differential diagnosis of haematuria [25]. Currently, no official national program for schistosomiasis exists [26]. The only government-run mass treatment program for schistosomiasis and soil-transmitted helminths in children was in KwaZulu-Natal Province during 1997–2000 [3]. There are comprehensive guidelines for treatment and control of schistosomiasis, which were expected to be implemented at district and provincial levels but they were not uniformly applied at primary healthcare facilities [3]. A national master plan for the elimination of neglected tropical diseases is being developed and includes a strategy for nation-wide interruption of schistosomiasis transmission but is not yet published. A number of barriers to praziquantel treatment have been reported. Treatment in South Africa is expensive because generic praziquantel is not registered by the Medicine Control Council even though it is accredited by the WHO [26]. The result is that costs per tablet are up to 40 times higher compared to other African countries, where the generic formulations are used [3,28]. A cost analysis of a study that treated 15 571 children in rural KwaZulu-Natal Province calculated the cost to treat each child as R50.88 (about US$ 3.30 currently) in 2012 [28]. This exorbitant cost has been cited as one of the reasons why many clinics in South Africa do not stock the drug [3,26]. Another barrier to treatment of children is the current formulation of praziquantel. The large tablet size and bitter taste make it difficult to swallow, and the 600 mg tablet does not allow for easy flexible dose adjustments needed for smaller children [29]. The high level of drug schedule for praziquantel in South Africa (schedule four) has been named as another limitation to treatment coverage of children in school-based mass drug administration programs. Since it must be administered by a healthcare professional, children absent from school on the day that the nurse came to dispense the drug, were not treated. This is in contrast to other countries where teachers assist with dispensing praziquantel at schools [3,28].

Most surveys using microscopic analysis to confirm the diagnosis recorded high prevalence estimates for schistosomiasis in endemic settings in South Africa between 2001 and 2017. For *S*. *haematobium*, prevalence of 24.5–98% was reported in schoolchildren in KwaZulu-Natal Province [12,17,30–32], 72% in schoolchildren in Eastern Cape Province [33] and 63–70% in rural villagers in Limpopo Province [34]. Lower prevalence was noted in younger age groups, with 1% [22] and 24% [32] in children aged 1–5 years in KwaZulu-Natal Province. Co-infection with *S*. *haematobium* and *S*. *mansoni* was recorded in 62–73% of schoolchildren sampled in Limpopo Province [35]. A cross-sectional study analysed stool samples collected in relation to diarrhoea from public hospital patients and schoolchildren in Limpopo Province and reported 14.4% prevalence of *S*. *mansoni* [36]. The prevalence for *S*. *mansoni* was lower in younger age groups with 0.9% reported among children aged 1–5 years in KwaZulu-Natal Province [22].

Even though schistosomiasis is known to be an important disease and undoubtedly causes morbidity and mortality [1,3,6,8,9], the standard treatment guidelines are not uniformly implemented and schistosomiasis control is of low priority among South African health authorities [3,37]. The lack of awareness of the true disease burden fuels further neglect. Effective control of the disease in South Africa is possible and has been demonstrated [3,32], but there is no national mass treatment strategy currently in place [3,22]. We used laboratory-based data to estimate and describe trends in the prevalence of microscopically-confirmed *S*. *haematobium* in South Africa.

## Methods

### Ethics statement

The Human Research Ethics Committee of the University of the Witwatersrand granted ethics approval for the National Institute for Communicable Diseases, a division of the National Health Laboratory Service, to collect and analyse data on communicable diseases in South Africa for surveillance purposes, and to share the findings with public stakeholders (clearance number M160667). Formal consent was not obtained because the data were anonymised.

### Study design

This is a descriptive analysis of microscopically-confirmed schistosomiasis and the testing patterns in South Africa. The study population included patients within the public health sector, confirmed to be infected with schistosomiasis by microscopic examination of urine or stool specimens, between 1 January 2011 and 31 December 2018.

### Data collection and processing

The National Health Laboratory Service (NHLS) has nationwide coverage and is responsible for all diagnostic laboratory testing within the public health sector of South Africa. The two datasets used in this study came from the LIS of the NHLS. The main dataset comprised patient records for all instances in which schistosomiasis was confirmed by microscopic examination of urine or stool specimens. The other dataset contained aggregated data for the numbers of times that urine specimens underwent microscopic parasitological examination, regardless of the outcome. We used this as an indicator of testing for *S*. *haematobium* in South Africa.

Deduplication of the patient records was done using the unique LIS patient number and date of birth in Stata version 15 (StataCorp, College Station, TX, USA). Records of patients that were retested within eight weeks of the first diagnosis were removed and any records with an implausible outcome were also deleted (e.g. *S*. *japonicum*, a species exotic to South Africa and microscopically distinct). The dataset containing testing numbers was deduplicated by the data analyst prior to being extracted from the data warehouse. Records with an age of zero were changed to missing if the date of birth was absent.

### Data analysis

#### Description of cases

Descriptive statistics were first applied to all cases of schistosomiasis (*S*. *haematobium* and *S*. *mansoni*) confirmed by microscopic testing during the analysis period.

#### Trends in prevalence

The numbers of stool specimens tested for *S*. *mansoni* could not be identified from the LIS data, so the main analysis was limited to *S*. *haematobium*. The national annual prevalence estimates for *S*. *haematobium* were calculated as a proportion of the total number of laboratory-diagnosed cases for each year out of the mid-year population estimate for that year, multiplied by 100 000. In the same way, the national annual test estimates were calculated by dividing the total number of urine tests performed each year by the mid-year population estimate for that year, multiplied by 100 000. The same method was used to produce sex-specific, age group-specific and province-specific prevalence and test estimates. Mid-year population estimates were obtained from the official government national census service, Statistics South Africa [38]. The confidence intervals for the proportions were calculated using Microsoft Excel (version 2016).

Trends in national annual prevalence and annual test estimates for *S*. *haematobium* were visualised with line graphs using Microsoft Excel (version 2016). The significance of the overall annual trends was assessed using simple linear regression in Stata version 15 (StataCorp, College Station, TX, USA). The outcome (annual prevalence/test estimate) and year variables made up the regression models. A slope that was not equal to zero (either increasing or decreasing) was only considered significant if the p-value was less than 0.05.

#### Patterns of testing

To appreciate the differences in testing for *S*. *haematobium* between different groups, we used a test rate ratio (TRR: Eq (1), below). Period prevalence estimates were calculated by dividing the total number of *S*. *haematobium* cases for the eight-year period by the population estimate for the middle of the study period (the mid-year estimate for the year 2014), multiplied by 100 000. Adjusted prevalence estimates were calculated by multiplying the period prevalence estimates with their respective TRR.


$$Test\;rate\;ratio\;\left(TRR\right)=\frac{test\;count\;(reference\;group)/population\;(reference\;group)}{test\;count\;(comparison\;group)/population\;(comparison\;group)}$$ (1)

The TRR was used to compare differences in testing between groups and is interpreted as any rate ratio. The reference group was chosen as the group with the highest period prevalence. Thus, if the TRR is greater than 1.0 it meant that the comparison group experienced less testing (relative to population size) than the reference group, and the effect was multiplicative. One would expect similar risk groups to be tested with similar rigor, i.e., the TRR for these groups should be close to 1.0 if one of them is the reference group.

## Results

### Schistosomiasis cases

Following the standard guidelines for selecting patients that should be tested based on clinical suspicion of schistosomiasis, 135 627 patients were diagnosed with schistosomiasis in 2011–2018. Table 1 gives an overview of all these cases. As shown, the predominant species detected was *S*. *haematobium* (99.8%, 135 372/135 627) and only 255 cases were *S*. *mansoni*. Where specimen type was specified, the most common specimen used to confirm schistosomiasis was urine (99.7%, 135 168/135 520), but some *S*. *haematobium* cases were detected by analysing stool. About 3% of all cases (4 466/135 627) had been diagnosed before, i.e., patients who tested positive again more than eight weeks after the previous diagnosis. These could be individuals who were never treated, individuals who were treated with the standard treatment (praziquantel) but remained infected, or those who became re-infected later after receiving effective treatment (Table 1). When aggregated by healthcare facility and visualised geographically (S1 Fig), the highest caseloads were observed in facilities along the eastern parts of the country. Although caseloads were lower, many facilities all over Gauteng Province and in the Cape Town area also recorded schistosomiasis. The proportion of urine specimens tested for *S*. *haematobium* that were positive was 11.1% (135 168/1 220 864).

**Table 1:** Characteristics of microscopically-confirmed schistosomiasis in the public sector, South Africa, 2011–2018.

Characteristic		Number (n)	Percentage (%)
**Species (N = 135 627)**	*Schistosoma haematobium*	135 372	99.8
	*Schistosoma mansoni*	255	0.2
**Specimen type (N = 135 520)**	Urine	135 168	99.7
	Stool	352	0.3
**First time diagnosis (N = 135 627)**	Yes	131 161	96.7
	No	4 466	3.3

### Trends in prevalence of *S*. *haematobium*

**Fig 1:**
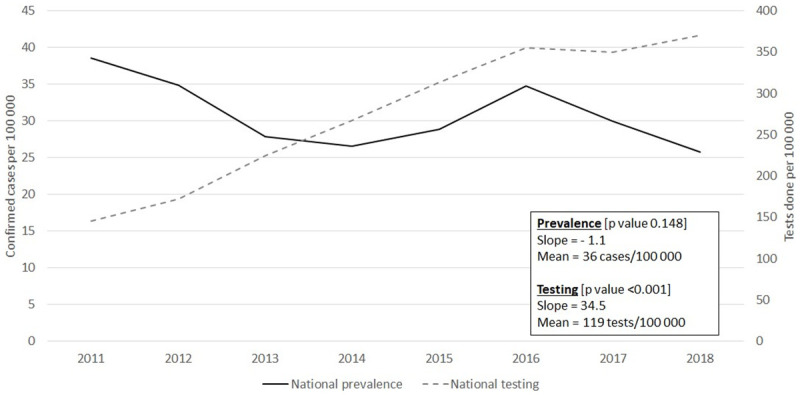
Trends in national annual prevalence estimates and testing for microscopically-confirmed *S*. *haematobium* in the public sector, South Africa, 2011–2018. The national prevalence estimate (solid black line) did not change substantially, while the national test estimate (grey dashed line) steadily increased each year. The mean, slope and statistical significance (p-value) of the linear trends are provided in the text box.

The annual trends in prevalence and testing for *S*. *haematobium* are demonstrated in Fig 1. There was a mean of 119 urine specimens tested per 100 000 persons per year, with an increasing trend of 35 tests per 100 000 persons per annum on average [slope = 35, p-value = <0.001]. There was no significant change in the national prevalence for schistosomiasis, with a mean of 36 cases per 100 000 persons per year [slope = -1.1, p-value = 0.148].

**Fig 2:**
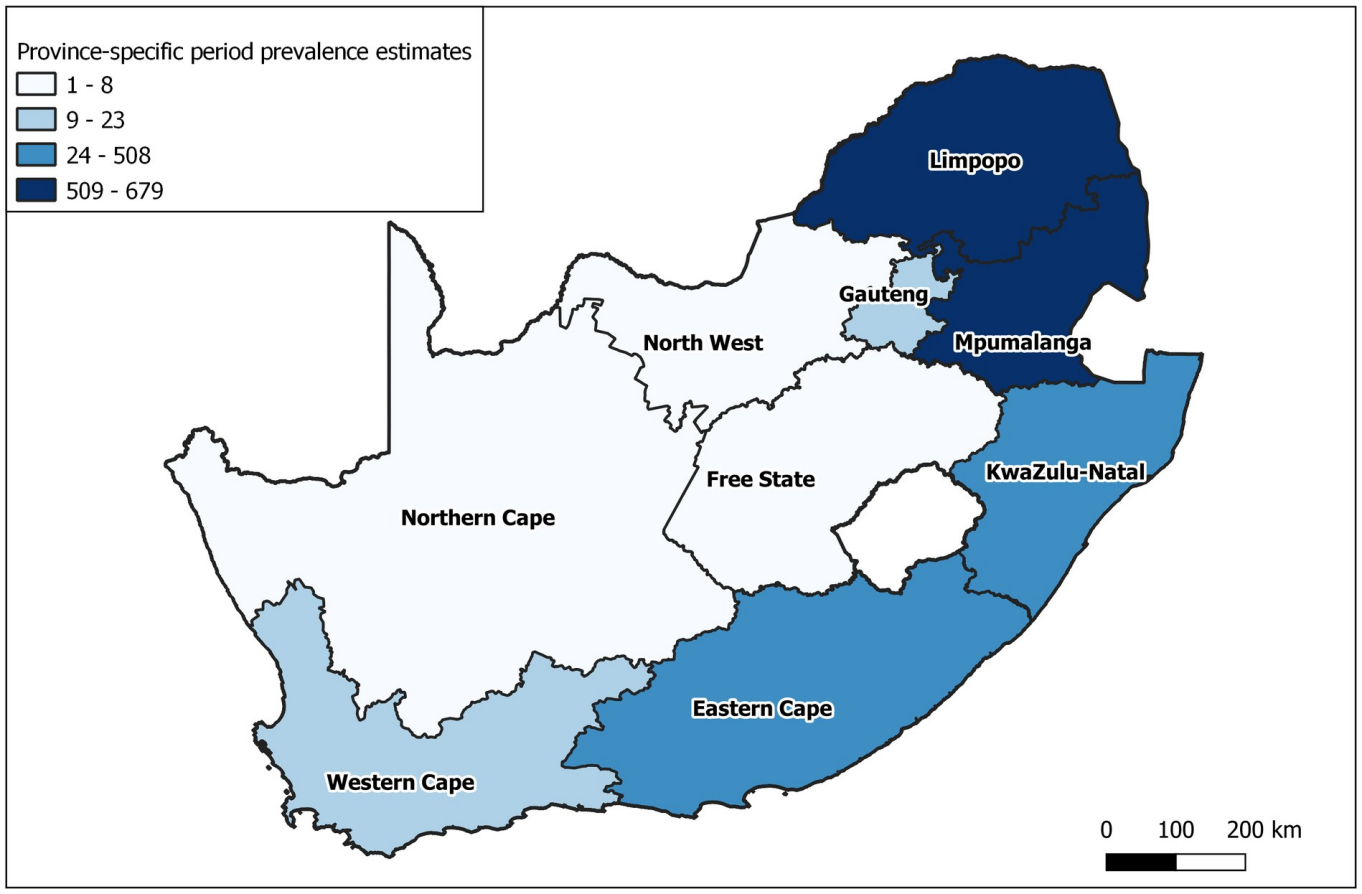
Province-specific period prevalence estimates of microscopically-confirmed *S*. *haematobium* in the public sector, South Africa, 2011–2018. Prevalence estimates are given per 100 000 persons and the intervals were selected using the Jenks natural breaks method. The map was made for this paper using Esri ArcGIS 10.2, with the 2016 provincial boundary shapefile from OCHA ROSEA under a Creative Commons Attribution for Intergovernmental Organisations (CC BY-IGO) license (https://data.humdata.org/dataset/south-africa-admin-level-1-boundaries).

The period prevalence estimates and TRR for *S*. *haematobium* are shown in Table 2. The period prevalence in males (348/100 000) was more than double that of females (141/100 000), and the age groups with the highest period prevalence were ages 5–19 years (ranging from 352 to 945 cases per 100 000). The endemic provinces of Limpopo, Mpumalanga and KwaZulu-Natal had the highest period prevalence (ranging from 507 to 679 cases per 100 000), followed by the provinces of Eastern Cape (141/100 000), Gauteng (23/100 000) and Western Cape (20/100 000). The choropleth map of period prevalence estimates is shown in Fig 2.

**Table 2:** Prevalence estimates and test rate ratios of microscopically-confirmed *S*. *haematobium* in the public sector, South Africa, 2011–2018.

Characteristic		Number of cases	Number of tests	Proportion positive tests (%)	Proportion positive tests (95% CI)	Mid-period Population estimate*	Period prevalence estimate (/100000)	Period prevalence estimate (95% CI)	Test rate ratio (TRR)	Adjusted prevalence estimate (/100000)	Adjusted prevalence estimate (95% CI)
**Sex**	Female	39 621	735 741	5.4%	5.3–5.4%	28 036 989	141	140–143	0.7	93	92–94
	Male	92 272	458 721	20.1%	20.0–20.2%	26 537 411	348	345–350	1.0 (ref)	348	345–350
	Not specified	3 479									
**Age group**	0–4	3 048	87 662	3.5%	3.4–3.6%	5 730 281	53	51–55	1.2	63	61–65
	5–9	19 089	57 953	32.9%	32.6–33.3%	5 424 838	352	347–357	1.7	598	593–603
	10–14	43 948	84 454	52.0%	51.7–52.4%	4 648 838	945	937–954	1.0 (ref)	945	937–954
	15–19	31 374	101 853	30.8%	30.5–31.1%	4 793 791	654	647–662	0.9	560	552–567
	20–24	17 541	138 766	12.6%	12.5–12.8%	5 327 010	329	324–334	0.7	230	225–235
	25–29	6 082	126 502	4.8%	4.7–4.9%	5 452 495	112	109–114	0.8	87	85–90
	30–39	3 197	28 695	11.1%	10.8–11.5%	8 371 164	38	37–40	5.3	202	201–204
	> = 40	2 826	347 086	0.8%	0.8–0.8%	14 825 983	19	18–20	0.8	15	14–15
	Not specified	8 267								0	
**Province**	Eastern Cape	9 385	118 113	7.9%	7.8–8.1%	6 634 413	141	139–144	1.1	157	155–160
	Free State	36	27 927	0.1%	0.1–0.2%	2 816 938	1	1–2	2.0	3	2–3
	Gauteng	3 112	219 415	1.4%	1.4–1.5%	13 376 960	23	22–24	1.2	28	27–29
	KwaZulu-Natal	54 177	468 078	11.6%	11.5–11.7%	10 686 971	507	503–511	0.5	229	225–234
	Limpopo	38 822	113 288	34.3%	34.0–34.5%	5 718 086	679	672–686	1.0 (ref)	679	672–686
	Mpumalanga	28 205	85 565	33.0%	32.6–33.3%	4 245 607	664	657–672	1.0	653	645–661
	Northwest	299	39 837	0.8%	0.7–0.8%	3 699 334	8	7–9	1.8	15	14–16
	Northern Cape	8	32 813	0.0%	0.0–0.0%	1 186 831	1	0–1	0.7	0	0–1
	Western Cape	1 259	115 828	1.1%	1.0–1.1%	6 209 261	20	19–21	1.1	22	20–23
	Not specified	69									
	South Africa	135 372	1 220 864	11.1%	11.0–11.1%	54 574 401	248	247–249	0.9	220	218–221

*Mid-year estimate for the year 2014.

### Comparison of testing for *S*. *haematobium*

Females experienced 30% higher testing compared to males (TRR = 0.7) within the NHLS. As expected, given their similar risk profiles, 15–19-year-olds were tested similarly to the 10–14-year-old reference group (TRR = 0.9), but 5–9-year-olds were relatively under-tested by about 70% (TRR = 1.7). The 0–4-year-old age group had a relative high testing intensity, almost the same as the 10–14-year-old group (TRR = 1.2). Among the endemic provinces, testing was similar in the two highest-prevalence provinces, Limpopo and Mpumalanga (TRR = 1.0), but testing in KwaZulu-Natal Province appeared to be much more, by about 50% (TRR = 0.5) compared to Limpopo Province. The TRRs calculated for the provinces of Eastern Cape, Western Cape and Gauteng were close to 1.0 (ranging 1.1 to 1.2), demonstrating similar testing as compared to the reference province of Limpopo.

To facilitate direct comparison, period prevalence estimates were adjusted using the TRR as weights to account for the differences in testing volumes between groups. None of the rankings within the population groups changed with this adjustment.

## Discussion

This description of microscopically-confirmed *S*. *haematobium* cases showed the highest burden of infection among people aged 5–19 years, male, and residents of Limpopo, Mpumalanga, and KwaZulu-Natal Provinces. Overall, the annual prevalence of *S*. *haematobium* in South Africa did not change substantially despite an overall increase in urine testing each year. Under-testing was demonstrated for the 5–9-year-old age group and higher testing was seen among females, children aged 0–4 years and residents of the KwaZulu-Natal Province, relative to their higher-burden counterparts.

Most of the literature on the prevalence of schistosomiasis refers to prevalence as a proportion of persons testing positive out of the number of persons tested, expressed as a percentage. The age groups with highest proportions of positive tests for *S*. *haematobium* observed in this study were 5–9 years (32.9%), 10–14 years (52.0%) and 15–19 years (30.8%). The literature on *S*. *haematobium* in South Africa reports similar age groups, especially 10–14 years, as having the highest burden. The prevalence ranged from 24.5% to 98% [12,17,30,32–34,39]. Although very few studies included age groups older than 15 years, one reported notable prevalence of at least 50% [40]. Variable prevalence of 1% [22] and 24% [32] was recorded in children aged 1–5 years in KwaZulu-Natal Province in contrast to the present study in which 3.5% of children aged 0–4 years were positive for *S*. *haematobium*. The proportion of positive tests for males (20.1%) was 3.7 times higher than females (5.4%) in this dataset. Many published South African studies reported no difference between males and females [11,12,35,40], but one also described higher prevalence in males [34]. The majority of literature on schistosomiasis prevalence in South Africa originates from KwaZulu-Natal followed by Limpopo and then Eastern Cape [11,12,15,17,30,32–36,40,41], reflecting these provinces’ historic higher burden of the disease. This was also seen in this dataset. The prevalence among Mpumalanga residents was similar to Limpopo in this study, suggesting that Mpumalanga Province is somewhat under-represented in the South African literature.

The prevalence of *S*. *haematobium* in South Africa remained consistent during the eight-year period, despite annual increases in testing. Thus, the unchanging prevalence could not be explained by decreased testing. In contrast, there was a significant global reduction of all-age deaths caused by schistosomiasis from 2007 to 2017 by 12.3% (95% CI 6.4%–17.6%) [6]. The decline in global deaths may have been a consequence of a drop in global prevalence, improved treatment and prevention, other changing environmental conditions, or differences in definitions for causes of death in the datasets used for the analysis. If the decline in global deaths was true, then it could be concerning that the prevalence in South Africa was unchanged. Nevertheless, constant prevalence during a time of absent large-scale mass drug administration and education campaigns in high-risk populations, is also an encouraging sign. It presents an opportunity to improve quality of life through implementation of control measures in high-burden areas or groups. There were likely multiple factors responsible for the steady disease prevalence observed including the consistency of suitable temperatures and habitats for the host snail population in South Africa during the eight-year period [42]. However, changes in temperatures and rainfall [43,44] and large-scale population movements or re-settlement could lead to changes in risk exposure to schistosomiasis in the future. Thus, there is still an urgent need for a national schistosomiasis control program.

Ideally, a successful schistosomiasis control program would follow the World Health Organization (WHO) guidelines while adapting to the South African setting, and should be improved over time as the needs change [45,46]. Long-term funding, political support and community involvement are vital for the sustainability of such a program [3,47]. Control programs should be incorporated into existing, low-cost integrated health packages and evaluated annually [45,47]. Several opportunities for implementing schistosomiasis control exist in South Africa. Since the National Department of Health adopted the primary healthcare approach in 2001, large-scale control programs have a better chance of effective implementation [32]. The guidelines for the new expanded program on immunisation recommend deworming for all children at appropriate ages [48] and treatment for soil-transmitted helminth infection by mass administration of deworming therapy to primary school children is currently in place [22,49]. Although this deworming programme treats solely soil-transmitted helminths, praziquantel could easily be added in appropriate areas. As the first government-run school-based parasite control program in KwaZulu-Natal Province showed during 1997–2000, successful local control of schistosomiasis in South Africa is feasible [12,32]. Valuable lessons about effective implementation of mass treatment campaigns in schools were learned by this program. These should be built upon within the new national master plan for the elimination of neglected tropical diseases, which includes schistosomiasis. These programs must reach the higher-burden population groups (males and ages 5–19 years), but in endemic settings they must also include the non-school going ages of 0–4 and 20–25 years to truly reduce morbidity in affected groups [22,50].

As expected, the highest burden of *S*. *haematobium* was found in the three provinces known to be endemic, but the prevalence in some other provinces warrants further discussion. First, our findings show that there was ongoing endemicity in the Eastern Cape Province during the period of analysis. The schistosomiasis epidemic profile in the Eastern Cape Province has always been dynamic, with long periods of apparent absence punctuated by periods of disease presence in the last century. Appleton and his colleagues [15] attributed this phenomenon to environmental and biological factors, saying that the host snails are sensitive to cold winters and dry conditions that may occur there. They classified the province as an area prone to periodic outbreaks [15]. We can only assume that favourable conditions prevailed during this period as we did not gather climatic data for this study. Second, the prevalence estimate of *S*. *haematobium* in Western Cape Province was higher than expected since transmission in this area is not known to occur [13–15]. It was similar to Gauteng Province, where transmission has been documented in the northern parts [13–16]. Both Gauteng and Western Cape provinces contain important metropolitan municipalities that attract regular economic migrants from South Africa’s rural areas. The cases detected in the Western Cape Province are more likely to have been imported rather than contracted locally. Using infection data such as these without parasite genetic information, it is impossible to say what proportion of the prevalence in Gauteng Province can be attributed to local transmission or importation. This is an important reminder that awareness of schistosomiasis is essential even in areas where the disease is not considered common, and highlights the need for heightened awareness among healthcare workers in metropolitan areas.

Urine testing volumes in some groups were discordant compared to their relative burdens of *S*. *haematobium*. Ideally, the 5–9-year-old age group should have been tested similarly to the 10–14-year-old age group, because their risk of infection is similar based on typical age-related behaviour. The fact that 0–4-year olds were tested more intensively than this group is interesting, since they are probably at a lower risk of infection, being less likely to swim in infested waters. However, these young children are still exposed to infection, perhaps during their mothers’ water-related activities, as reflected by the 3.5% positivity proportion observed in the 0–4-year old age group in this dataset and a study in rural KwaZulu-Natal Province [22]. Similarly, males were shown to experience 2.5 times higher burden of infection and yet females were tested more. It is probable that the higher level of interaction with healthcare providers by women and children aged 0–4 years produced this apparent bias in testing volumes. Nevertheless, it may represent resources that could be better directed towards higher risk groups. It is hoped that this paper will increase awareness of these high-burden groups that require special attention regarding this disease. The high frequency of testing in KwaZulu-Natal Province is a promising sign. Again, the lessons learned and the experience gained in KwaZulu-Natal Province could be valuable and should be shared with other endemic provinces to further improve their testing numbers.

Our findings contribute to the existing knowledge of the prevalence in South Africa and represent an updated report of nationwide *S*. *haematobium* prevalence, to complement the 1980 Atlas of Bilharzia in South Africa [14]. Since the disease was classified as a NMC in 2017 [21], the reporting system for NMCs has been re-engineered. The surveillance and monitoring system was to be rolled out across the country from 2018 to 2020. Going forward, awareness of the NMCs may grow as the new platforms for reporting are launched and with the new system being easier to use, reporting might increase. Our findings contribute to the baseline prevalence of schistosomiasis prior to it becoming a NMC.

A major strength of this study is that it is the first time that nationally representative patient-level data could be analysed for schistosomiasis. Although the data came from the public health sector only, most South Africans (ranging 73–87%) access this sector for healthcare purposes [24]. Thus, we believe that these findings may be generalised to the South African population, particularly those at high risk. The study was limited by the reliance on laboratory data and their nature. The patient records captured in the LIS relied on the testing strategies of the healthcare workers. We used the records of urine specimens tested for parasites as the denominator to estimate the prevalence proportions. Since the urine tests were likely requested due to a higher level of suspicion (presence of haematuria or other symptoms), it could have led to an overestimation of the proportions. This may also help to explain some of the less logical testing patterns seen. For example, higher testing was noted for women and young children, who also interact more with healthcare providers. To give balance to this bias in the proportions of positive tests, the prevalence estimates were also reported as cases per 100 000 of the population. The numbers of stool specimens tested for *S*. *mansoni* could not be identified from the LIS data, so the main analysis was limited to *S*. *haematobium*. Only the variables collected by the laboratory were available with no other clinical or therapeutic information, which would have enriched the dataset. Although the data were probably representative, we may have underestimated prevalence because early cases could have been missed since serological tests were excluded, the patients may not have been excreting eggs yet, not all cases seek healthcare to be tested, and some cases may be treated empirically without being tested at all. We used the place of diagnosis (the healthcare facility) as the location, rather than the place of transmission because home addresses were not reliably provided in the data. This might have been somewhat inaccurate, but according to a general household survey most South African households use the healthcare facility closest to their home [24] so it was considered an acceptable indicator for place of transmission. The reliability of the test results may have been influenced by the sensitivity and specificity of microscopic testing, which probably varied between laboratories and technologists. However, this bias was present for all records, in all locations. Finally, when the group-specific prevalence estimates were adjusted using the TRR, a linear relationship between test volume and prevalence was assumed, and this may not have been true for all of the groups, particularly non-endemic provinces.

In conclusion, we found that schistosomiasis remains a major public health concern in South Africa, and we demonstrated that laboratory-based passive surveillance could be a practical tool for reporting prevalence, and for monitoring and evaluating future intervention programs. Policies and interventions should be targeted at young people and males especially in those high-burden endemic areas and metropolitan areas that receive imported cases. To reduce the cost of treatment, the South African government should prioritise registration and licensing of generic praziquantel formulations, and down-scheduling of the drug would facilitate easier and more cost-effective dispensing [3,26,28]. There are many gaps in the knowledge of schistosomiasis in South Africa that require further investigation. The lack of a denominator for *S*. *mansoni* testing limited this analysis. Other important areas for study include costs and implementation strategies for mass administration of praziquantel, current environmental risk assessment and the role of animal schistosome species.

## Supporting information

S1 Fig Locations and facility-level caseloads of microscopically-confirmed schistosomiasis in the public sector, South Africa, 2011–2018. Showing absolute case numbers per facility, the highest caseloads were detected at healthcare facilities along the eastern regions of the country. The map was made for this paper using Esri ArcGIS 10.2, with the country boundary shapefile from openAFRICA under a Creative Commons Attribution (CC BY 4.0) license (https://open.africa/dataset/africa-shapefiles) and the 2016 provincial boundary shapefile from OCHA ROSEA under a Creative Commons Attribution for Intergovernmental Organisations (CC BY-IGO) license (https://data.humdata.org/dataset/south-africa-admin-level-1-boundaries).
(TIF)
