# Supplementary figures and images for: Trends in the prevalence of microscopically-confirmed schistosomiasis in the South African public health sector, 2011–2018
Source: PLoS Negl Trop Dis. 2021 Sep 16;15(9):e0009669. doi: 10.1371/journal.pntd.0009669 (PMC8445405; doi:10.1371/journal.pntd.0009669)

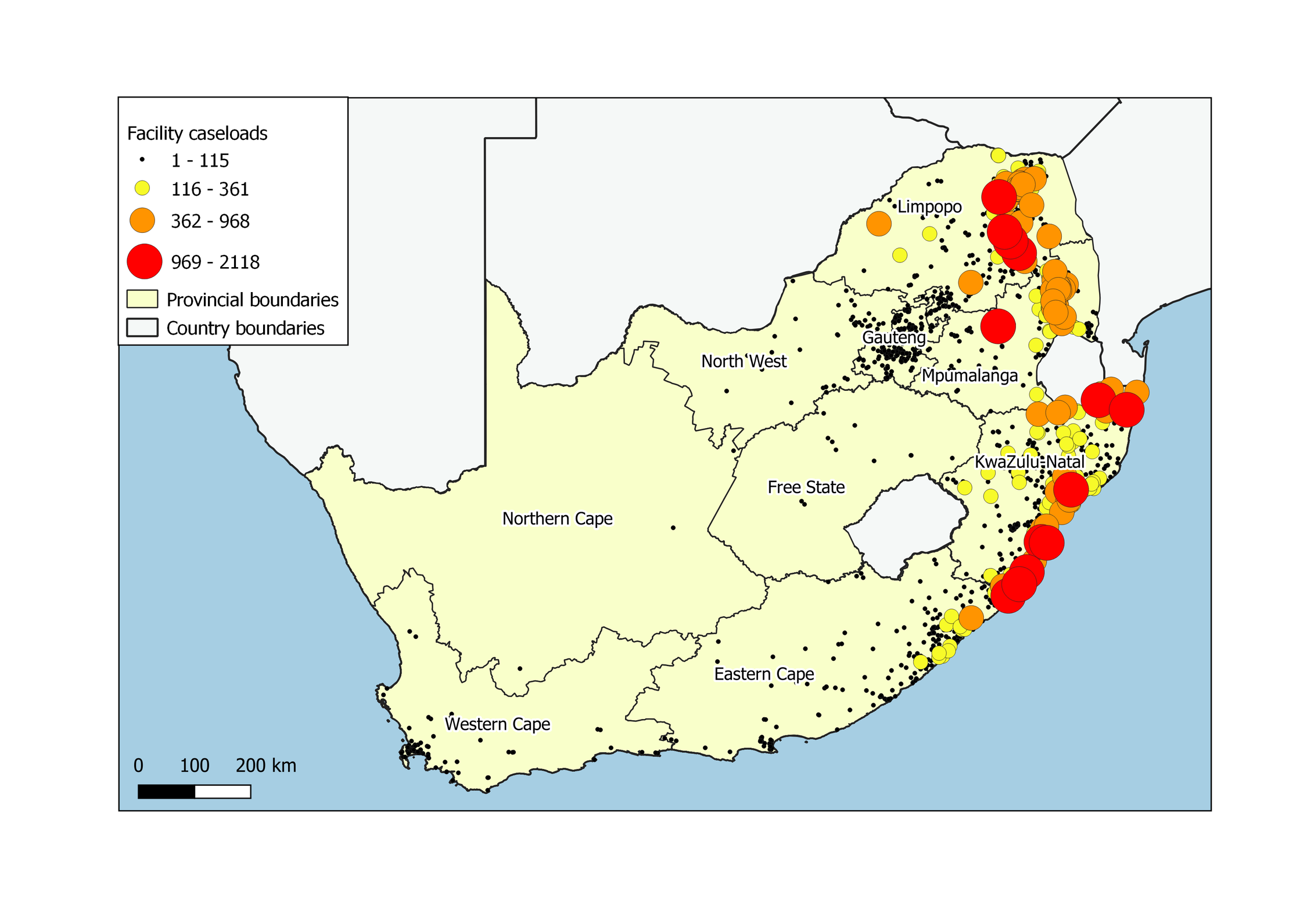

Supplement: S1 Fig — Showing absolute case numbers per facility, the highest caseloads were detected at healthcare facilities along the eastern regions of the country. The map was made for this paper using Esri ArcGIS 10.2, with the country boundary shapefile from openAFRICA under a Creative Commons Attribution (CC BY 4.0) license (https://open.africa/dataset/africa-shapefiles) and the 2016 provincial boundary shapefile from OCHA ROSEA under a Creative Commons Attribution for Intergovernmental Organisations (CC BY-IGO) license (https://data.humdata.org/dataset/south-africa-admin-level-1-boundaries). (TIF) [file pntd.0009669.s001.tif]
